# The Y-Worri Project: study protocol for a randomised controlled trial

**DOI:** 10.1186/1745-6215-14-76

**Published:** 2013-03-19

**Authors:** Alison L Calear, Helen Christensen, Kathleen M Griffiths, Andrew Mackinnon

**Affiliations:** 1 Centre for Mental Health Research, The Australian National University, Canberra, Australia; 2 Black Dog Institute, University of New South Wales, Sydney, Australia; 3 Orygen Research Centre, University of Melbourne, Melbourne, Australia

**Keywords:** Adolescent, Anxiety, Prevention, School, Online

## Abstract

**Background:**

Anxiety disorders are one of the most common psychological problems in adolescents. The school system has been identified as an ideal setting for the implementation of prevention and early intervention programs for anxiety; however, few programs are routinely delivered in schools and little is known about the best delivery methods. The aim of the current project is two-fold: to test the effectiveness of an intervention program for anxiety relative to a control condition, and to compare two methods of implementing the program.

**Methods/design:**

This study is a three-arm cluster randomised controlled trial consisting of a wait-list control condition and two intervention conditions evaluating the effectiveness of an Internet-based program for preventing generalised anxiety. The first intervention condition will involve classroom teachers supervising student completion of the intervention program, while the second intervention condition will involve the classroom teacher and an education officer from the local youth mental health centre supervising the program’s completion. At least 30 schools from across Australia will be recruited to the trial, with adolescents aged between 14 and 18 years invited to participate. Participants in the intervention conditions will complete the e-couch Anxiety and Worry program during class periods over six weeks. The primary outcome measure will be a scale reflecting the number and severity of generalised anxiety symptoms, while secondary outcomes will be symptoms of depression, social anxiety and anxiety sensitivity. Data will be collected at pre-intervention, post-intervention, 6- and 12-month follow-up. Intention-to-treat analyses will be conducted.

**Discussion:**

If demonstrated effective, a new service delivery model for the implementation of mental health programs in schools could be indicated. Such a model would significantly contribute to the mental health of young people in Australia by providing preventive interventions for mental health problems and consequently reducing the need for clinical services.

**Trial registration:**

The Australian New Zealand Clinical Trials Registry ACTRN12610001103055

## Background

Anxiety disorders are one of the most common psychological problems in adolescents and young adults and often precedes the development of depression [1,2]. The 1-year prevalence rate of anxiety disorders in this population is estimated to lie between 5% and 21% [3-6], with 8% to 27% of young people experiencing an anxiety disorder during adolescence [3-5,7]. The effect of anxiety disorders on the wellbeing of young people is substantial, with their social, emotional and academic functioning often severely affected [8]. If left untreated, the effects of anxiety typically continue into adulthood where further occupational, economic and interpersonal difficulties can arise [9].

Given the high prevalence rate of anxiety disorders and their associated negative effects, efforts directed at prevention and early intervention should be paramount. Preventing the onset or worsening of anxiety symptoms during adolescence would not only reduce and prevent the short- and long-term effects described above, but could also contribute to reducing the need, and thus cost, of clinical treatment [10], and potentially prevent the onset of depressive disorders [11].

The school system has been identified as an ideal setting for the implementation of prevention and early intervention programs for anxiety, due to its unparalleled contact with youth [12,13]. The delivery of anxiety intervention programs in schools is also important given the low rates of disorder identification [14,15] and help-seeking behaviour by adolescents [16,17]. Many young people, particularly men, do not seek help for anxiety. This was reflected in the 2007 National Survey of Mental Health and Wellbeing which found that only 13.2% of young men aged between 16 and 24 years with a mental disorder had sought help [18].

A number of effective school-based prevention and early intervention programs for anxiety have been developed and implemented [19,20]. These programs tend to target current anxiety levels with the aim of preventing anxiety symptoms from worsening or preventing the development of anxiety symptoms at all. Despite their effectiveness, these programs are not routinely delivered in schools. The reasons for the lack of uptake of effective programs are not clear. It is apparent however, that teachers are often not keen to deliver mental health programs in the classroom due to a lack of training or general expertise in the field [21]. Teachers may be anxious about the effect of mental health materials on their students, and may be concerned that they themselves will be unable to handle any consequences. These problems indicate an important need to develop appropriate methods of delivering and implementing effective anxiety intervention programs in schools, particularly if schools are to function as a vector for mental health symptom reduction and prevention. A major concern has also been in the perception that behavioural change needs to be maintained, and that appropriate channels to seek help when required are needed in a comprehensive model.

The aims of the present project, therefore, are two-fold. The first is to test the effectiveness of an online intervention program for anxiety relative to a control condition. The second is to determine whether an enhanced version of the program which incorporates access to a potential service provider results in improved outcomes relative to the standard teacher administered intervention. The anxiety intervention to be evaluated in the current trial is an automated anxiety literacy and cognitive behavioural therapy (CBT) program delivered via the Internet. This program has the potential to overcome some of the problems associated with traditional face-to-face school-based programs by providing a self-directed program to students that requires little teacher training or knowledge. The automated delivery of Internet-based interventions also ensures the fidelity of a program, while its self-directed nature can promote independent learning.

The use of online programs in schools for the prevention of depression has been found to be effective in the trials to date [22-24]. The YouthMood Project [24], which evaluated the MoodGYM program in 30 schools from across Australia in a universal prevention trial, found significant reductions in depressive symptoms in men (Cohen’s *d* = 0.27-0.43), and in anxiety symptoms in men and women (*d* = 0.15-0.25). Earlier controlled trials of the MoodGYM program in schools found similar effects [22,23].

Two delivery methods will be compared to the control condition, in which students will continue their usual classes during the intervention phase of the trial. The first implementation method to be tested in the current trial will involve classroom teachers supervising student completion of the Internet-based program for reducing and preventing anxiety in schools (‘e-GAD school method’ condition). The second implementation method adds the support and assistance of *headspace* to the first approach (‘e-GAD health service method’ condition). *headspace* is an Australian youth mental health foundation which provides mental health services and support for young people aged 12 to 24 years. *headspace* will guide students in the completion of the program and provide support to students if and when they disclose personal difficulties, or require further help and support. *headspace* will also promote the mental health services that they provide and raise student awareness of the help-seeking options available. This condition will ascertain whether *headspace* assisted delivery differs in effectiveness from that of the teacher delivered intervention.

### Aims of the project

#### Primary aim

(1) To evaluate the efficacy of the e-couch Anxiety and Worry program in reducing and preventing symptoms of anxiety in an adolescent school-based population relative to usual school classes (wait-list control). Both the teacher delivered and *headspace* assisted conditions will be compared to the wait-list control condition.

#### Secondary aims

(1) To compare classroom delivered e-couch with *headspace* assisted e-couch to determine whether the addition of *headspace* contact in the classroom improves outcomes.

(2) To evaluate the effect of the e-couch Anxiety and Worry program (teacher delivered or *headspace* assisted) on depressive symptoms, wellbeing, anxiety literacy, anxiety stigma, and help-seeking attitudes, intentions and behaviour compared to the wait-list control condition.

(3) To assess participant satisfaction, attendance and adherence to the e-couch Anxiety and Worry program, and to determine the predictors of these outcomes, and their relationship to mental health outcomes.

(4) To evaluate the effect of the e-couch Anxiety and Worry program on individuals with and without clinical levels of anxiety or depressive symptoms at pre-intervention.

#### Hypotheses

(1) The e-couch Anxiety and Worry program (whether delivered by the school or the health service method) will be associated with significantly lower levels of generalised anxiety symptoms at post-intervention, 6- and 12-month follow-up, than the wait-list control condition.

(2) The effect of the e-couch Anxiety and Worry program on anxiety symptoms and help-seeking behaviour will be greater in the guided e-GAD health service method than in the e-GAD school method.

(3) The e-couch Anxiety and Worry program (delivered by either method) will be associated with significantly lower levels of depressive symptoms and anxiety stigma, and higher levels of wellbeing, anxiety literacy and help-seeking attitudes, intentions and behaviours at post-intervention, 6- and 12-month follow-up, than participants in the wait-list control condition.

(4) The e-couch Anxiety and Worry program (delivered by either method) will prevent the development of anxiety and depressive symptoms over 12 months in those who are asymptomatic at baseline.

## Methods

### Study design

This study will be implemented as a three-arm cluster stratified randomised controlled trial (RCT) with two interventions conditions (e-GAD school method condition and e-GAD health service method condition) and a wait-list control condition. There will be four measurement occasions: pre-intervention, post-intervention, 6- and 12-month follow-up. This study was granted ethical approval by the Australian National University Human Ethics Committee (protocol number 2010/550) and the individual Sate and Territory Education Departments responsible for each participating school.

### Recruitment

At least 30 schools from across Australia will be recruited to participate in the trial. Schools will be drawn from rural and metropolitan areas and male and female adolescents in grades 9 to 12 (aged 14 to 18 years) will be invited to participate. Schools will be recruited according to the location of participating *headspace* centres. Information letters will be sent to each school informing them of the project. A briefing day will be held for all participating schools prior to the trial’s implementation. The briefing day will provide schools with an opportunity to meet other schools involved in the trial and to receive detailed information and instruction on the delivery of the trial. Information and consent forms will be distributed to all invited participants and their parents/guardians, with written informed consent required from both.

### Randomisation

Each participating school (cluster) will be randomised to one of the three conditions. Randomisation will occur according to *headspace* centre location. Cluster randomisation will be employed for administrative convenience, to avoid wait-list control condition contamination, and for the ecological validity of providing the intervention at the cluster level [25,26]. A statistician not involved in the implementation of the trial will randomly allocate schools within each *headspace* centre to the intervention or wait-list control conditions using a computerised random number generator. The identity of schools will be concealed from the statistician during this process.

### Procedure

All participating students will be invited to complete a pre-intervention, post-intervention, 6- and 12-month follow-up questionnaire. Participants randomised to the intervention conditions will undertake the e-couch Anxiety and Worry program (http://www.ecouch.anu.edu.au) during one class period a week (30 to 40 min) for 6 weeks following the pre-intervention questionnaire. All participating students will be provided with an e-couch Anxiety and Worry program login and password, which will be linked to their questionnaire ID. This will allow program adherence and completion rates (which are recorded automatically by the e-couch Anxiety and Worry program) to be linked to anxiety and other variable outcomes. Individual sections of the e-couch Anxiety and Worry program will be made available to participants each week to prevent participants completing the program prematurely. The e-couch Anxiety and Worry program will be incorporated into the school curriculum and will be delivered in a range of subject areas, including pastoral care, health, personal development and English.

Classroom teachers in the e-GAD school method condition will supervise students’ completion of the e-couch Anxiety and Worry program, with no direct teaching. Participants exhibiting elevated levels of anxiety and/or depression will be referred to the school counsellor in accordance with usual school procedures. School counsellors will be provided with clear referral pathways to the local *headspace* centre if further support is required.

Classroom teachers in the e-GAD health service method condition will supervise students’ completion of the e-couch Anxiety and Worry program, with the assistance and support of *headspace* centre staff. h*eadspace* centre staff will provide a brief description of the *headspace* service, and the contact details of the local *headspace* centre. In the classroom they will respond to student questions about the program and offer referral pathways for students who request help. The *headspace* centre staff will also act as an external source of contact for students who may not feel comfortable approaching school staff for assistance. Essentially the *headspace* centre staff will guide students in completion of the program and promote the importance of help-seeking behaviour for mental health problems.

Schools in the wait-list control condition will continue their usual classes during the intervention phase of the trial. The activities undertaken by participants in the wait-list control condition will be dependent on the class in which the trial is implemented. Participants in the wait-list control condition schools will be offered the e-couch Anxiety and Worry program at the conclusion of the trial (following the 12-month follow-up questionnaire). All classroom teachers will be provided with a step-by-step guide to assist them in the administration of the trial questionnaires and the delivery of the e-couch Anxiety and Worry program.

### Intervention

The e-couch Anxiety and Worry program (http://www.ecouch.anu.edu.au) consists of two major parts: psychoeducation and a series of evidence-based toolkits for anxiety consisting of CBT, relaxation and physical activity. The psychoeducation section is modelled on mental health literacy interventions previously found to improve attitudes and reduce symptoms for depression and anxiety [27,28]. The content of the psychoeducation section is as follows: definition of worry; distinction from stress; differentiation of worry, fear and anxiety; description of anxious thinking; differentiation of GAD from panic disorder (PD), specific phobia and social anxiety disorder (SAD), adjustment disorder and post-traumatic stress disorder (PTSD); description of risk factors for GAD; the problem of co-morbidity; consequences of anxiety; and treatments for anxiety including medical, psychological and lifestyle treatments. The content of these sections is based on reviews of evidence of alternative and lifestyle treatments [29] and on clinical practice guideline [28].

The CBT toolkit for anxiety is based on previously developed material with confirmed efficacy for anxiety cognitions and beliefs in at-risk individuals [30,31]. This toolkit teaches participants about worry, what causes and compounds worry, how to detect and reduce worry, how to problem solve the issues causing worry, how to change thoughts to prevent and reduce worry, and exploring the feelings behind worry. This toolkit essentially focuses on the cognitive aspects of worry and how to change them. The vast majority of school-based prevention programs for anxiety are based on CBT techniques such as these and have been found to be both efficacious and safe [19,20].

The relaxation toolkit contains a mindfulness meditation exercise and progressive muscular relaxation exercise. In the mindfulness meditation exercise participants are encouraged to focus on their breathing and to be in the present moment, rather than lost in thoughts, memories or the future. Mindfulness meditation promotes a sense of calm. Progressive muscular relaxation involves tensing and relaxing the different muscle groups in the body. This exercise helps participants to recognise the tension in their bodies and how to relieve it.

The physical activity toolkit was designed to help participants evaluate their own level of physical activity, learn some strategies for increasing or maintaining their physical activity level, and teach them some of the benefits of being physically active. The toolkit recommends 30 min of physical activity a day, which includes partaking in day-to-day activities such as hanging out the washing, gardening and walking up stairs. The toolkit does not focus on strenuous physical activity and participants who are not currently physically active are advised to start slowly.

### Assessments

Table 1 presents the scales that will be administered at each measurement occasion in the Y-Worri Trial.

**Table 1:** Questionnaire scales for the Y-Worri trial

	**Pre-intervention**	**Post-intervention**	**6-month follow-up**	**12-month follow-up**
Demographics	X			
Anxiety history	X			
Self-perceived emotional health	X	X	X	X
SCAS	X	X	X	X
GAD-7	X	X	X	X
SAS-A	X	X	X	X
CASI	X	X	X	X
CES-D	X	X	X	X
WEMWBS	X	X	X	X
GASS (Personal)	X	X	X	X
GASS (Perceived)	X			
Predictors adherence	X	X	X	X
Mental health literacy	X	X	X	X
Anxiety literacy (A-Lit)	X	X	X	X
Days out of role	X	X	X	X
ATSPPH-SF	X	X	X	X
Adapted GHSQ	X	X	X	X
Adapted AHSQ	X	X	X	X
Usefulness of e-couch		X	X	X
Use of e-couch strategies		X	X	X
Helpfulness of e-couch strategies		X	X	X

AHSQ, Actual Help-Seeking Questionnaire; ATSPPH-SF, Attitudes Toward Seeking Professional Psychological Help- Short Form; CASI, Childhood Anxiety Sensitivity Index; CES-D, Center for Epidemiological Studies Depression Scale; GASS, Generalised Anxiety Stigma Scale; GHSQ, General Help-Seeking Questionnaire; SAS-A, Social Anxiety Scale for Adolescents; SCAS, Spence Children’s Anxiety Scale; WEMWBS, The Warwick-Edinburgh Mental Well-being Scale.

#### Demographic variables

The following demographic variables will be measured: age, sex, grade, rural location, first language, Aboriginal or Torres Strait Islander origin, living situation, and participant history of anxiety and help-seeking behaviour.

#### Generalised anxiety disorder

Generalised anxiety disorder (GAD) symptoms will be the primary outcome of interest for the study and will be measured on the Spence Children’s Anxiety Scale (SCAS) [32] and the GAD-7 [33]. An effect size of approximately 0.3 is expected at both post-intervention and follow-up. The expected 0.3 effect size is further discussed in the power calculations. The SCAS is a 44-item self report questionnaire composed of 38 items that assess specific anxiety symptoms relating to six subscales, namely generalised anxiety, social phobia, separation anxiety, panic attack/agoraphobia, obsessive-compulsive disorder and physical injury fears, and six positive ‘filler items’ designed to reduce negative response bias. Each item is responded to on a four point scale ranging from 0 (never) to 3 (always). A total scale score is calculated by summing the scores for the 38 anxiety symptom items. The total scale score on the SCAS ranges from 0 to 114, with higher scores reflecting greater levels of anxiety. The GAD-7 is a brief seven-item self-report scale that measures generalised anxiety symptoms. Each item is responded to on a four-point scale ranging from 0 (not at all) to 3 (nearly every day). A total scale score is calculated by summing item scores, with total scale scores ranging from 0 to 21. Higher GAD-7 scores indicate greater levels of generalised anxiety symptoms. An additional eighth item will also be included to assess the impact of anxiety symptoms, if present, on functioning.

#### Social anxiety

The Social Anxiety Scale for Adolescents (SAS-A) [34] will be employed in the current study to measure social anxiety symptoms. The SAS-A is a 22-item measure that consists of 18 descriptive self-statements assessing the respondent’s subjective experience of social anxiety, and four filler items reflecting activity or social preferences. Each item is rated on a five-point scale ranging from 1 (not at all) to 5 (all the time). A total scale score can be obtained by summing the ratings for the 18 descriptive self-statements, with total scores ranging from 18 to 90. Higher SAS-A scores are indicative of greater social anxiety symptoms. Three subscales can also be derived from the SAS-A which measure fear of negative evaluation (FNE), social avoidance and distress with new social situations or unfamiliar peers (SAD-New), and more generalised social avoidance and distress (SAD-General).

#### Anxiety sensitivity

The Childhood Anxiety Sensitivity Index (CASI) [35] will be administered in the current study to measure symptoms of anxiety sensitivity, which is associated with panic attacks and panic disorder. The CASI consists of 18 items that assess the extent to which the respondent believes that the experience of anxiety will result in negative consequences. Each item is responded to on a three-point scale ranging from 1 (none) to 3 (a lot). Total scale scores are calculated by summing the ratings across all items. CASI scores can range from 18 to 54, with higher scores reflecting greater levels of anxiety sensitivity.

#### Depressive symptoms

Depressive symptoms will be measured on the Center for Epidemiological Studies Depression Scale (CES-D) [36], which consists of 20 items. Respondents indicate the frequency with which they have experienced symptoms during the past week on a four-point Likert type scale ranging from 1 (rarely or none of the time (<1 day)) to 4 (most or all of the time (5 to 7 days)). A total scale score is calculated by summing items scores, after reverse scoring those items that are negatively worded. Total scale scores can range from 0 to 60, with higher scores indicative of more depressive symptomatology.

#### Wellbeing

Mental wellbeing will be assessed by the Warwick-Edinburgh Mental Well-being Scale (WEMWBS) [37]. The WEMWBS is a self-report scale that consists of 14 positively worded items that measure different aspects of positive mental health. Each item is rated on a five-point Likert type scale ranging from 1 (none of the time) to 5 (all of the time), with a total scale score calculated by summing item scores. Total WEMWBS scores can range from 14 to 70, with higher scores indicating a greater level of mental wellbeing.

#### Anxiety stigma

Personal and perceived anxiety stigma will be measured by the Generalised Anxiety Stigma Scale (GASS) [38], which consists of two parallel sets of items. The first set of 10 items is designed to assess the respondent’s personal attitudes to stigma (GASS-Personal); the second set of 10 items measure the respondent’s perception of the attitudes of others (GASS-Perceived). The respondent is asked to rate each item on a five-point scale ranging from 0 (strongly disagree) to 4 (strongly agree). Total GASS-Personal and GASS-Perceived scores are calculated by summing the respective item scores, with total scale scores ranging from 0 to 40. Higher total scale scores are indicative of greater personal or perceived anxiety stigma.

#### Anxiety literacy

Anxiety literacy (A-Lit) [39] was assessed using a 22-item test of anxiety knowledge (for example, sign and symptoms, causes, treatments), which was adapted from the previously developed depression literacy scale (D-Lit) [27]. Each item on the A-Lit is rated on a ‘true/false/don’t know’ scale and is scored by assigning a 1 to each correct response and a score of 0 to each incorrect or ‘don’t know’ response. A total scale score is calculated by summing the item scores. Total scale scores can range from 0 to 22, with higher scores reflecting greater anxiety literacy. We will also assess attitudes to treatments by asking the respondent to rate whether each of the interventions listed are likely to be helpful, harmful or neither for someone with anxiety. This measure was also adapted from a previous measure for depression [40].

#### Help-seeking

Help-seeking attitudes, intentions and behaviours will be measured using the Attitudes Toward Seeking Professional Psychological Help-Short Form (ATSPPH-SF) [41] and an adapted version of the General Help-Seeking Questionnaires (GHSQ) and Actual Help-Seeking Questionnaire (AHSQ) [42]. The ATSPPH-SF is a 10-item measure of attitudes toward seeking psychological help. Each item is responded to on four-point Likert type scale ranging from 0 (disagree) to 3 (agree). A total scale score is calculated by summing items scores, after reverse scoring those items that are negatively worded. Total scale scores can range from 0 to 30, with higher scores indicative of more positive attitudes toward seeking psychological help.

The adapted version of the GHSQ assesses intentions to seek help from 14 different sources (for example, friend, mother, psychologist, teacher) and 13 treatments or activities (for example, CBT, meditation, Internet-based program) for anxiety. Respondents indicate how likely they are to seek help from or use each of the sources or treatments on a scale ranging from 1 (extremely unlikely) to 7 (extremely likely). The adapted AHSQ assesses recent help-seeking behaviour and consists of the same 14 sources of help and 13 treatments or activities for anxiety, which the respondent either does or does not report seeking help from.

#### Disability

Disability caused by anxiety symptoms will be assessed using a four-item open-ended measure of the number of full and partial days out of role in the past 30 days due to anxiety.

#### Satisfaction

Participant satisfaction with the e-couch Anxiety and Worry program will be measured through an assessment of individual usage of the program and its perceived usefulness using measures developed for previous ANU online trials. Example items will include ‘How much did you learn from the website?’, ‘Do you think you will use the website in the future?’ and ‘Would you recommend the website to others?’ Participant use, and the perceived helpfulness, of the strategies and techniques taught in the e-couch Anxiety and Worry program will also be individually assessed, as will participant knowledge and use of *headspace* mental health services.

#### Adherence

Participant adherence to the program will be measured by the number of program modules and exercise completed, which will be automatically recorded by the software of the e-couch Anxiety and Worry program. A number of potential predictors of adherence will also measured in the current study. These will include the perceived efficacy of Internet-based interventions (for example, ‘I am confident that people could learn skills for preventing anxiety from a website’), Internet literacy (for example, ‘I am confident in my ability to use the Internet’), motivation (for example, ‘rate the importance of preventing an episode of clinical anxiety over the next year’), and behavioural intentions (for example, ‘I want to change the way I think and feel about emotional problems’).

### Sample size and power calculations

Assuming an average class size of 25, a total of 750 students will be available in 30 classes. Based on our previous work [24], we anticipate an intraclass correlation (ICC) of 0.02 and non-participation rate of 30%, with 5% of this non-participation/completion arising from students leaving the school or being absent on measurement occasions. The majority of non-participation (estimated at 25%) will result from failure to return parental consent forms resulting in an inability to participate in the study. In the current study, we plan to address this problem by using electronic communication with parents. We anticipate that this will result in data from 525 students being available. The ICC of 0.02 leads to a design effect of 1.48 and an effective sample size of 354. Assuming a correlation between measurements over time of 0.5, two-tailed tests and alpha of .05, the trial would have 80% power to detect group differences down to 0.3 standard deviations. A group difference of 0.3 standard deviations is marginally higher than the effects obtained using MoodGYM as a universal intervention but, as the current intervention is specifically aimed at anxiety, a slightly larger effect can be reasonably expected. The magnitude of the incremental effect arising from adding *headspace* support to the e-GAD school method condition is uncertain. Comparison of the school method and health service method conditions could be conceptualized within a non-inferiority framework [43] however the sample size required using a defensible non-inferiority margin would be unfeasible. Given the additional costs and resources associated with providing health service support, it was considered acceptable to power the trial to be able to detect differences between the two active conditions comparable in magnitude to comparisons with the wait-list control condition.

### Statistical analysis

Primary analyses of the outcomes will be undertaken on an intent-to-treat basis, including all participants randomised regardless of treatment actually received or withdrawal from the study. Mixed models repeated measures (MMRM) analyses will be used because of the ability of this approach to include participants with missing data. This approach will also allow potential clustering effects (students within the same school being more alike) to be explored and accounted for within the analyses. If required appropriate transformations of scores will be undertaken. Should outliers or distributional problems prelude standard methods, generalized mixed models will be applied. A series of analyses will be conducted to assess the effectiveness of the e-couch Anxiety and Worry program in preventing and reducing symptoms of generalised anxiety. These analyses will include a general analysis of intervention effects comparing the two delivery method conditions to the wait-list control condition, as well as separate analyses to test the effectiveness of the individual delivery methods. MMRM analyses of the secondary variables (for example, depressive symptoms, anxiety stigma) will also be conducted.

Subsample analyses will also be conducted with participants who scored above and below the clinical cutoff scores for anxiety and depressive symptoms at pre-intervention. The preventive subsample analyses will be conducted to identify the true preventive effects of the intervention, while the clinical subsample analyses will be undertaken to provide valuable information regarding the utility of the e-couch Anxiety and Worry program as an indicated intervention. MMRM ANOVAs, identical to those applied to the full sample, will be conducted on the four subsamples of participants. In addition to this, analyses will be conducted to identify any significant differences in the proportion of participants in the intervention and wait-list control conditions who meet criteria for clinical caseness at post-intervention, 6- and 12-month follow-up in the preventative sub-sample analyses, or remain clinical cases in the clinical subsample analyses (attributable risk reduction). The number needed to treat (NNT) will be calculated with 95% confidence intervals (CIs).

## Discussion

In Australia, as in most countries, very few prevention programs for mental health are implemented in schools outside of the trial context, even though there is evidence for their effectiveness. The aim of the current project is to address this problem by trialling two implementation methods that support the delivery of these programs by teachers in the classroom. If demonstrated effective, a new service delivery model for the implementation of mental health prevention programs in schools could be introduced nationally. Such a model would significantly contribute to the mental health of young Australians by providing preventive interventions for mental health problems and consequently reduce the need for clinical services.

Approximately 600,000 young Australians experience a mental health problem. Based on population estimates, and a fully implemented universal program, it is likely that school-based intervention programs could reduce this rate by approximately 14% to 21% [44] thereby conservatively reducing the number with a disorder by as many 90,000. To be feasible, these programs need to be conducted by school teachers. Unlike other school-based programs, the active component of this intervention is delivered automatically online and does not require the training of teachers or the use of specialist psychologists. To our knowledge, this will be one of the first intervention trials for anxiety delivered primarily through the web in school environments. It is also the first trial to compare the effectiveness of different methods of delivering the program, and it has specific and applied implications for prevention delivery across Australia.

## Trial status

Participant recruitment was completed in June 2012. Data collection is ongoing and is expected to be completed in June 2013.

## Competing interests

HC and KMG are the authors and developers of the e-couch website but derive no personal or financial benefit from its operation.

## Authors’ contributions

All authors wrote the funding application to the Vincent Fairfax Family Foundation and contributed to the design of the study. ALC drafted the manuscript. All authors contributed to the editing of the manuscript and approved the final manuscript.
